# Genetic variability in a Brazilian apple germplasm collection with low chilling requirements

**DOI:** 10.7717/peerj.6265

**Published:** 2019-01-21

**Authors:** Livia Costa Mariano, Felipe Liss Zchonski, Clandio Medeiros da Silva, Paulo Roberto Da-Silva

**Affiliations:** 1 Plant Genetics and Molecular Biology Laboratory, Universidade Estadual do Centro-Oeste, UNICENTRO, Guarapuava, Paraná, Brazil; 2 Instituto Agronômico do Paraná, Londrina, Paraná, Brazil

**Keywords:** Genetic divergence, Pre-breeding, *Malus*, Inter-simple sequence repeat, ISSR, Molecular markers

## Abstract

The apple (*Malus domestica* Borkh) originally evolved to require temperatures below 7.2 °C for the induction of budding and flowering. In Brazil, breeders have overcome the climate barrier and developed the cultivars Anabela, Julieta, Carícia, and Eva, with low chilling requirements and good yield characteristics. These cultivars are grown in many warmer climate countries in South America, Africa, and the Middle East. The apple germplasm collection that originated these cultivars has several genotypes with pedigrees for a low chilling requirement. Knowledge of the variability and genetic relationships among these genotypes may be useful in the development of superior new cultivars. In this work, we first selected the best ISSR (inter-simple sequence repeat) primers for genetic studies in apple, and then we used the selected primers to evaluate the genetic variability of the apple germplasm collection at the Instituto Agronômico do Paraná. The evaluation of 42 ISSR primers in 10 apple genotypes allowed us to select the best nine primers based on the polymorphic information content (PIC) and resolving power (RP) indexes. The primer selection step was robust since the dendrogram obtained with the nine selected primers was the same as the one obtained using all 26 polymorphic primers. Primer selection using PIC and RP indexes allowed us to save about 60% of time and costs in the genetic variability study. The nine ISSR primers showed high levels of genetic variability in the 60 apple genotypes evaluated. The relevance of the primer selection step is discussed from the perspective of saving time and money in germplasm characterization. The high genetic variability and the genetic relationships among the genotypes are discussed from the perspective of the development of new apple cultivars, mainly aiming for a low chilling requirement that can better adapt to current climatic conditions or those that may arise with global warming.

## Introduction

The apple (*Malus domestica* Borkh) is a species of the family Rosaceae, subfamily Pomoideae, which comprises 100 genera and around 2,000 species spread throughout the world (Folta & Gardiner, 2009; Hofer et al., 2014). The center of origin of the apple is Kazakhstan and Central Asia; more specifically, the genus *Malus* primarily originated in Asia Minor, the Caucasus, Central Asia, the Indian Himalayas, Pakistan, and Western China, where there are at least 25 to 47 native *Malus* species (Yan et al., 2008; Folta & Gardiner, 2009; Hofer et al., 2014).

The apple was originally a temperate tree, growing where winters are cold (with average temperatures of −5 °C) and summers are mild (with average temperatures of 15 °C). Currently, the species is also cultivated in subtropical regions with less rigorous winters (0 to 10 °C), and even in tropical climates, such as in Northeast Brazil (Pommer & Barbosa, 2009). In these regions, the apple shows problems with climatic adaptation (Lopes et al., 2012).

Since the beginning of apple cultivation in Brazil, several breeding programs have been initiated by official research institutions such as the Instituto Agronômico de Campinas (IAC) in São Paulo, Instituto Agronômico do Paraná (IAPAR) in Paraná, the Epagri in Santa Catarina, and the Embrapa, in Rio Grande do Sul (Pommer & Barbosa, 2009). Several cultivars have been released by these breeding programs (for review, see Pommer & Barbosa, 2009), with the IAC breeding program being the most active until the 1990s. Owing to the colder climate of southern Brazil, the production area expanded rapidly and the quality of apples produced in this region overlapped with those of the apples produced in São Paulo. This led to a drastic decline in the planted area in this state after the 1990s, with the interest in research on new cultivars declining correspondingly (Hauagge & Bruckner, 2002).

The apple breeding program at IAPAR, a government research institution of Paraná State, began in 1979 (Hauagge & Bruckner, 2002). The main objectives of this program are the development of cultivars with low chilling requirements (LCR), high yields, early maturation, high fruit quality, and resistance to apple scab (Pommer & Barbosa, 2009). Among the cultivars that have been released by IAPAR are Eva, Anabela, Carícia, and Julieta, all of which have LCR (<500 chilling units) (Hauagge & Tsuneta, 1999). For apples, a chilling unit is equal to one hour of a plant exposure to chilling temperatures. Chilling temperatures extend from freezing point to 7.2 °C. Eva is a low chill (250–400 chilling units required) and early fruiting cultivar, capable of producing high quality fruit in warmer areas where apples have previously not been grown (Pommer & Barbosa, 2009). This cultivar was developed from the cross between the cultivars Anna and Gala (Hauagge & Tsuneta, 1999). In Brazil, Eva is widely adapted and is still cultivated from the south (Rio Grande do Sul state) to the tropics (Pernambuco state). It is also grown in similar climates in many countries of South America, Africa, and the Middle East (Castro et al., 2015; Pommer & Barbosa, 2009).

Apple production in Brazil is actually concentrated in the Southern Region, i.e., the states of Rio Grande do Sul, Santa Catarina, and Paraná, and within smaller areas in the states of Minas Gerais, São Paulo, Bahia, and Pernambuco (Pommer & Barbosa, 2009; IBGE, 2012). The main cultivars produced in Brazil are Gala and Fuji (Pommer & Barbosa, 2009). The breeding program in Rio Grande do Sul, Santa Catarina, and Paraná states are still active. However, LCR cultivars were not the main objectives of the breeding programs in Santa Catarina and Rio Grande do Sul states due to the subtropical climate of these states. On the other hand, since the state of Paraná has a warmer climate, the search for apple cultivars with low chilling requirements is the main objective of the IAPAR breeding program.

The apple breeding program of IAPAR maintains a germplasm collection with approximately 200 genotypes, including traditional cultivars and genotypes obtained by crosses performed by this institution. Among these genotypes, there is a great interest in the genotypes related to the cultivars Eva, Anabela, Julieta, and Carícia, which require low chilling units to flower, are resistant to apple scab, and have good market characteristics (Pommer & Barbosa, 2009). The use of biotechnology to aid field evaluations may help breeders to identify these genotypes and plan promising crosses to obtain materials with greater adaptation to warm regions.

Among the molecular techniques, the use of molecular markers has been highlighted as an aid to breeders, whether for the characterization of germplasm collections or even for assisted selection. Among molecular markers, the use of the ISSR (inter-simple sequence repeat) marker is simple, efficient, and inexpensive. These markers, with dominant inheritance, are based on the amplification of regions between the microsatellite sequences in the genome. The ISSR markers were developed to address the need to explore the large amount of variation in the repetitive regions of the genome without the need for large-scale sequencing (Zietkiewicz, Rafalski & Labuda, 1994). The amplicons are another important feature of the ISSR markers, because they range from 200 to 2,000 bp in length, facilitating gel analysis (Bornet & Branchard, 2001; Reddy, Sarla & Reddy, 2002). These markers show high reproducibility when compared with other nonspecific PCR-based markers, such as the *random* amplified polymorphic DNA (RAPD) (Wolfe & Liston, 1998).

Since the IAPAR apple germplasm collection has not yet been characterized with molecular markers, ISSR can be a good tool to use for this purpose. Therefore, this work aimed to select, from among 42 ISSR primers, the most robust ones for genetic studies in apple, and to use them to resolve the genetic relationship between the apple genotypes of the IAPAR germplasm collection to assist in the selection of genotypes for future crosses in order to obtain new cultivars with low chilling requirements and good production and market characteristics.

## Material and Methods

### Plant material, DNA extraction and molecular analysis

For this study, 60 apple genotypes (Table 1) were selected from the IAPAR apple germplasm collection kept at an experimental station in Palmas county, PR, Brazil (26°27′56″S 51°58′33″W). These genotypes include commercial cultivars and genotypes that have shown good characteristics and performance in the field. Four young leaves of each genotype were collected and kept on silica gel until DNA extraction.

**Table 1:** Codification and pedigree of apple genotypes from IAPAR germplasm collection evaluated in this work.

**Codification**	**Genotype**	**Pedigree**
1	31 − 80 − 5	Mollie’s Delicious × Anna
2	52 − 80 − 2	Dei Argentina × Anna
3	9 − 80 − 12	Granny Smith × Anna
4	13 − 80 − 1	Freyberg × Anna
5	303 − 81 − 27	no information
6	30 − 80 − 63	Willie Sharp × Anna
7	78 − 80 − 11	no information
8	28 − 80 − 66	Golden Delicious × Anna
9	EVA	Anna × Gala
10	284 − 81 − 21	no information
11	307 − 81 − 7	no information
12	1 − 80 − 168	Prima × Anna
13	1 − 80 − 255	Prima × Anna
14	MALUS-44	Selected by Epagri
15	26 − 80 − 77	Super Golden Spur × Anna
16	15 − 80 − 24	Red Spur × Anna
17	27 − 80 − 54	Gala × Anna
18	85 − 80 − 12	no information
19	MALUS-16	Selected by Epagri
20	310 − 81 − 9	no information
21	53 − 80 − 154	P x 1033 OPS^a^
22	6 − 80 − 28	Golden Delicious × Anna
23	28 − 80 − 14	Golden Delicious × Anna
24	2 − 80 − 33	Gala × Anna
25	284 − 81 − 26	no information
26	53 − 80 − 59	P × 1033 OPS^a^
27	1 − 80 − 146	Prima × Anna
28	2 − 80 − 54	Gala × Anna
29	1 − 80 − 277	Prima × Anna
30	28 − 80 − 28	Golden Delicious × Anna
31	1 − 80 − 185	Prima × Anna
32	4 − 80 − 15	Super Golden Spur × Anna
33	1 − 80 − 5	Prima × Anna
34	26 − 80 − 53	Super Golden Spur × Anna
35	38 − 80 − 1	WinterBanna × Anna
36	2 − 80 − 166	Gala × Anna
37	IMPERIAL GALA	Mutation from Gala
38	CARÍCIA	Anna × Prima
39	27 − 80 − 2	Gala × Anna
40	ANABELA	Anna × Gala
41	JULIETA	Anna × Mollie’s Delicious
42	FUJI SUPREMA	Mutation from Fuji
43	2 − 80 − 202	Gala × Anna
44	2 − 80 − 7	Gala × Anna
45	25 − 80 − 77	Golden Delicious × Anna
46	34 − 80 − 3	Belgonden × Anna
47	2 − 80 − 59	Gala × Anna
48	31 − 80 − 34	Mollie’s Delicious × Anna
49	1 − 80 − 165	Prima × Anna
50	303 − 81 − 44	no information
51	172 − 88 − 304	Eins Shemmer OPS
52	8 − 80 − 5	Royal Red Delicious × Anna
53	14 − 80 − 5	Red Delicious × Anna
54	1 − 80 − 35	Prima × Anna
55	302 − 81 − 205	no information
56	25 − 80 − 59	Golden Delicious × Anna
57	26 − 80 − 144	Super Golden Spur × Anna
58	ANNA	Red Hadassiya × Golden Delicious
59	53 − 80 − 149	P × 1033 OPS^a^
60	CASTEL GALA	Mutation from Gala

**Notes.**

a Selection from the progeny originating from the open pollinated PX 1033.

Before DNA extraction, the leaves were ground in liquid nitrogen to obtain a fine powder. The DNA was extracted following the protocol proposed by Doyle & Doyle (1987). The extracted DNA was quantified by electrophoresis on 0.9% agarose gel stained with ethidium bromide (0.5 µg mL^−1^). To determine the amount of DNA in each sample, comparisons were made with known amounts of Phage *λ* DNA (50, 100, 200, and 400 ng).

To select the best ISSR primers for genetic studies in apple, 42 primers from the UBC (University of British Columbia, Vancouver, Canada) series (Table 2), were evaluated in 10 apple genotypes (307_81_7, 1_80_168, 1_80_255, 26_80_77, 15_80_24, 85_80_12, MALUS_16, 310_81_9, 53_80_154, and 28_80_28).

**Table 2:** The 42 ISSR primers used, along with their respective sequences and parameters in *Malus domestica* Borkh. Annealing temperature in °C (AT°C), amplification product (AP), total number of amplified fragments (TN), percentage of polymorphism (%P), polymorphism information content (PIC), and resolving power (RP). The shaded primers were the nine most informative ones in *Malus*.

**Primer**	**Sequence^a^**	**AT°C**	**AP**	**TN**	**%P**	**PIC**	**RP**
UBC 807	(AG)_8_T	52	✓	20	45.00	0.34	3.00
UBC 808	(AG)_8_C	50	–	–	–	–	–
UBC 809	(AT)_8_T	55	–	–	–	–	–
UBC 810	(GA)_8_T	52	–	–	–	–	–
UBC 811	(GA)_8_C	53	–	–	–	–	–
UBC 813	(CT) _8_T	50	✓	9	44.44	0.36	2.00
UBC 814	(CT)_8_A	50	✓	9	22.22	0.33	1.00
UBC 815	(CT)_8_G	53	✓	11	81.81	0.42	6.20
UBC 817	(CA)_8_A	52	✓	10	70.00	0.39	4.00
UBC 820	(GT)_8_T	52	✓	7	57.14	0.44	2.60
UBC 822	(TC)_8_A	55	✓	12	25.00	0.29	1.40
UBC 823	(TC)_8_C	55	✓	13	30.76	0.43	2.80
UBC 824	(TC)_8_G	50	–	–	–	–	–
UBC 826	(AC)_8_C	52	✓	11	54.54	0.40	4.00
UBC 827	(AC)_8_G	53	✓	11	9.09	0.30	0.80
UBC 828	(TG)_8_A	50	–	–	–	–	–
UBC 834	(AG)_8_YT	52	✓	18	38.88	0.45	5.40
UBC 835	(AG)_8_YC	54	✓	12	33.33	0.42	2.40
UBC 836	(AG)_8_YA	53	✓	13	30.76	0.47	3.20
UBC 840	(GA)_8_YT	53	–	–	–	–	–
UBC 843	(CT)_8_RA	54	✓	12	66.66	0.37	4.20
UBC 848	(CA)_8_RG	55	–	–	–	–	–
UBC 852	(CT)_8_RA	52	✓	6	50.00	0.41	2.00
UBC 855	(AC)_8_YT	55	✓	14	21.42	0.47	2.40
UBC 856	(AC)_8_YA	55	✓	4	50.00	0.46	1.60
UBC 857	(AC)_8_YG	54	✓	12	33.33	0.44	2.00
UBC 858	(TG)_8_RT	52	✓	8	50.00	0.37	2.00
UBC 859	(TG)_8_RC	55	–	–	–	–	–
UBC 860	(TG)_8_RA	52	✓	6	83.33	0.37	2.40
UBC 861	(ACC)_6_	52	–	–	–	–	–
UBC 864	(ATG)_6_	50	–	–	–	–	–
UBC 866	C(TCC)_5_TC	55	–	–	–	–	–
UBC 868	(GGA)_6_	50	✓	22	13.63	0.39	2.00
UBC 873	(GACA)_4_	50	✓	16	31.25	0.40	3.00
UBC 878	GGA(TGGA)_3_T	54	–	–	–	–	–
UBC 881	(GGGGT)_3_	53	✓	9	33.33	0.37	1.80
UBC 886	VDV(CT)_7_	55	–	–	–	–	–
UBC 889	DBD(AC)_7_	52	✓	6	83.33	0.18	0.40
UBC 890	VHV(GT)_7_	54	✓	11	9.09	0.32	0.40
UBC 891	HVH(TG)_7_	54	✓	10	30.00	0.37	1.60
UBC 899	CATGGTGTTGGTCATTGTTCCA	55	–	–	–	–	–
UBC 900	ACTTCCCCACAGGTTAACACA	55	–	–	–	–	–

**Notes.**

a Y = (C,T); R = (A, G); H = (A, C, T); B = (C, G, T); V = (A, C, G); D = (A, G, T).

PCR and electrophoresis followed the protocols proposed by Rosa et al. (2017).

### Statistical analysis for ISSR primer selection

Only fragments showing good resolution patterns were considered. The amplicons were scored visually for the presence (1) or absence (0) of the fragments being analyzed, creating a binary matrix. To select the best ISSR primers based on the binary matrix obtained, the discriminatory power of each primer pair was evaluated using polymorphism information content (PIC) and resolving power (RP).

The PIC for each ISSR loci was computed following Roldan-Ruiz, Dendauw & Vanbockstaele (2000). The RP was calculated according to Prevost & Wilkinson (1999).

To identify the best primers for genetic variability studies in apples, the primers were first organized according to the highest values of each of the two parameters. The primers that showed the best PIC and RP values were selected.

The Jaccard similarity coefficient was calculated using the NTSYS 2.2 software (Rohlf, 2000) and the similarity dendrogram among the apple genotypes was obtained by the Unweighted Pair Group Method Using Arithmetic Averages (UPGMA) method. The robustness of the primer selection was evaluated by comparative analysis of the dendrogram obtained with nine selected primers, with the dendrogram obtained from all polymorphic primers. The reliability of branches in the dendrograms was assessed by bootstrapping with 1,000 replicates.

### Genetic variability data

To evaluate the genetic variability of the 60 genotypes of the IAPAR apple germplasm collection using the nine most informative primers, PCR, electrophoresis, and gel evaluation were performed as described in the ISSR primer selection section.

Genetic similarity among the genotypes was estimated by Jaccard’s similarity coefficient using the NTSYS 2.2 software (Rohlf, 2000). After obtaining the matrix, the average similarity between all the genotypes was calculated and the dendrogram was obtained by the UPGMA method.

Principal Coordinates Analysis (PCoA) was performed using NTSYS 2.2 (Rohlf, 2000) and GenAlex (Peakall & Smouse, 2012). To obtain genetic groups, Bayesian analysis was performed using the software STRUCTURE (Pritcharda, Wena & Falushb, 2010). To determine the ideal number of genetic groups (K), simulations were performed based on the assumption that it is possible to obtain any number of genetic groups from 1 to 10, where each simulation was repeated 10 times. For this analysis the allelic frequencies were correlated by 1,000 burn-in and 10,000 Markov Chain Monte Carlo (MCMC) repeats after the burn-ins. The Structure Harvester program (Earl, 2012) was used to determine the more probable K values than those indicated by the analysis criteria suggested by Evanno, Regnaut & Goudet (2005).

## Results

### Selection of ISSR primers for genetic studies in apple

Of the 42 ISSR primers tested in 10 apple genotypes, 26 (61.90%) showed amplification with good fragment resolution (Table 2). These 26 primers amplified 292 fragments, with an average of 11.23 fragments per primer. Of these fragments, 113 (38.69%) were polymorphic. The primer UBC 856 showed the lowest number of amplified fragments (4), while the primer UBC 868 showed the highest number (22). The size of the fragments ranged from 210 bp (UBC 873) to 1600 bp (UBC 856). The average polymorphism per primer ranged from 9.09% (UBC 827 and UBC 890) to 83.33% (UBC 860 and UBC 889).

The PIC values calculated in this study ranged from 0.18 (UBC 889) to 0.47 (UBC 836), with an average of 0.38. The RP ranged from 0.40 (UBC 889 and UBC 890) to 6.20 (UBC 815), with an average of 2.48.

The best (highest) values of PIC and RP were used to select the nine most informative primers (Table 2): primers UBC 815, UBC 817, UBC 823, and UBC 834, were selected based on the PIC values; and primers UBC 807, UBC 826, UBC 836, UBC 843, and UBC 873, were selected based on the RP values.

**Figure 1:**
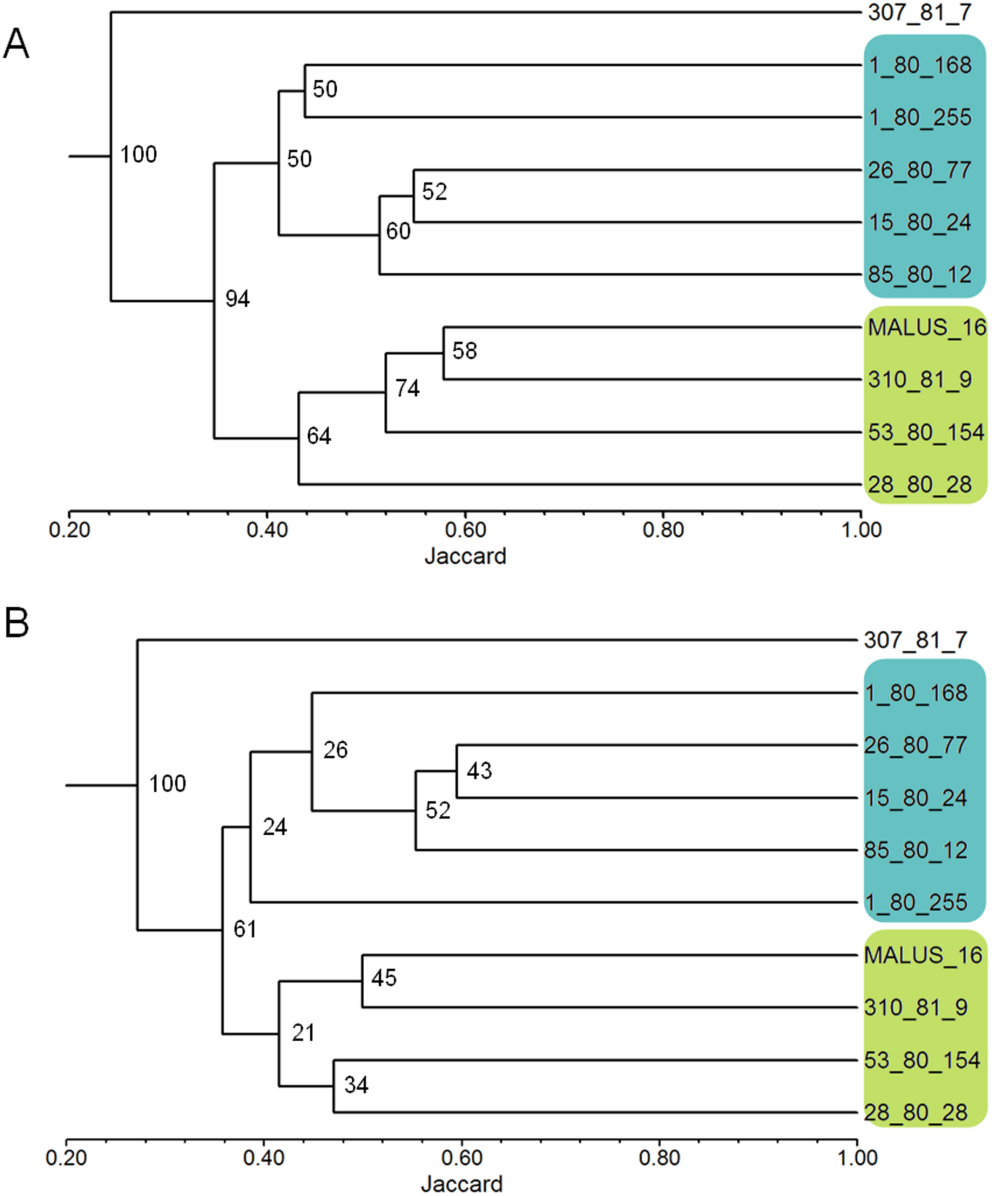
Dendrograms of 10 apple genotypes obtained with the data from the 26 ISSR primers (1A) and with the nine ISSR primers selected as the best for genetic studies in *Malus* (1B). The numbers on the branches indicate the bootstrapping values obtained with 1,000 replicates.

The robustness test of the selected primers showed that the clustering of the 10 apple genotypes using the data from all 26 polymorphic primers and the clustering obtained using the nine selected primers were very similar (Figs. 1A and 1B). The genetic similarity obtained with data from the 26 primers ranged from 0.14 to 0.49, with an average of 0.37 among all genotypes; and with data from the nine selected primers, the similarity ranged from 0.16 to 0.54, with the same average value of 0.37.

### Genetic variability of the Brazilian apple germplasm collection

**Figure 2:**
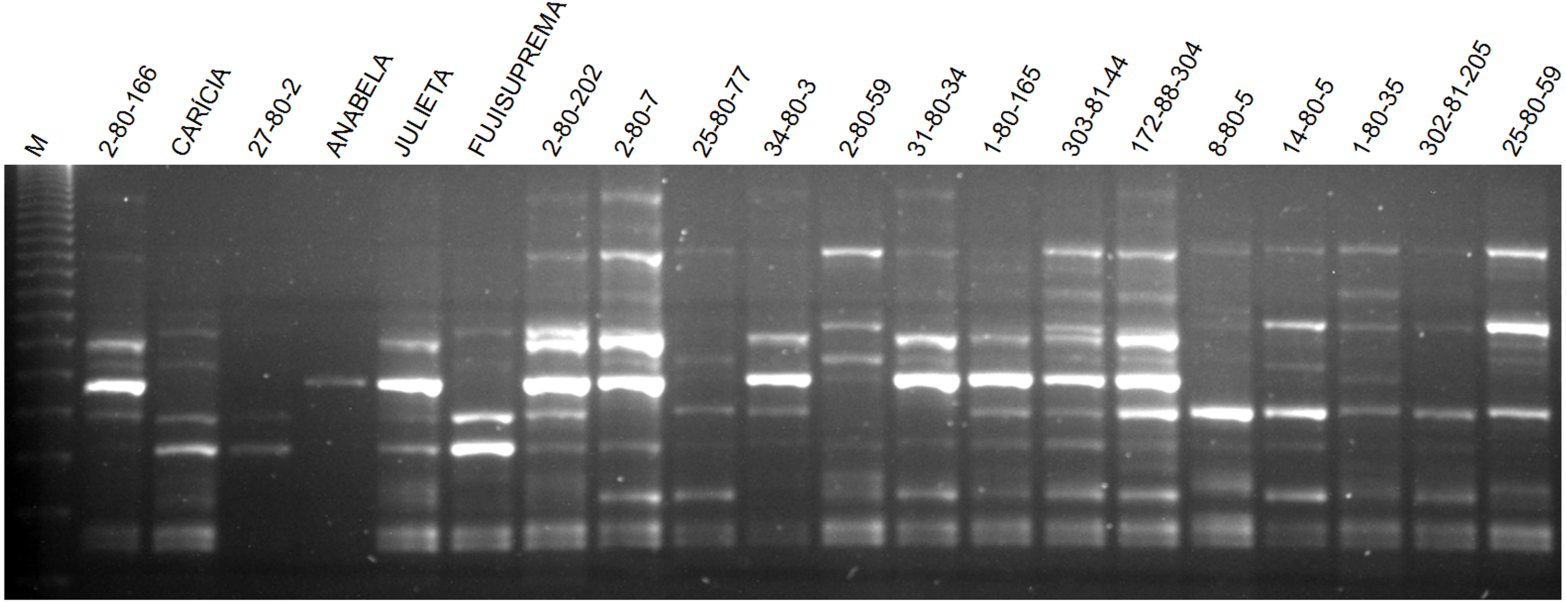
Agarose gel with the amplification pattern of primer ISSR 807 in some apple genotypes evaluated in this work. M indicates the 123 bp molecular weight marker.

The total number of fragments amplified by the nine ISSR primers in the 60 apple genotypes was 129, with an average of 14.33 fragments per primer. Seventy-five of these fragments were polymorphic, with an average of 8.3 polymorphic fragments per primer. The primer UBC 807 had the highest number of amplified fragments and the primer UBC 843 had the highest number of polymorphic fragments as well as the highest percentage of polymorphism (Table 3). The average polymorphism of the nine primers in the 60 apple genotypes was 58.1%. The pattern of ISSR markers amplification in apple is shown in Fig. 2.

**Table 3:** The nine ISSR primers used to estimate the genetic variability of 60 apple genotypes from the IAPAR germplasm collection.

**Primer**	**AT °C**	**TNAF**	**NFP**	**% P**
UBC 807	52	28	15	53.5
UBC 815	52	18	11	61.1
UBC 817	52	10	5	50.0
UBC 823	52	13	5	38.4
UBC 826	52	10	4	40.0
UBC 834	52	11	6	54.5
UBC 836	53	13	8	61.5
UBC 843	54	16	14	87.5
UBC 873	50	10	7	70.0

**Notes.**

AT annealing temperature in °C TNAF total number of amplified fragments NPF number of polymorphic fragments %P percentage of polymorphism

**Figure 3:**
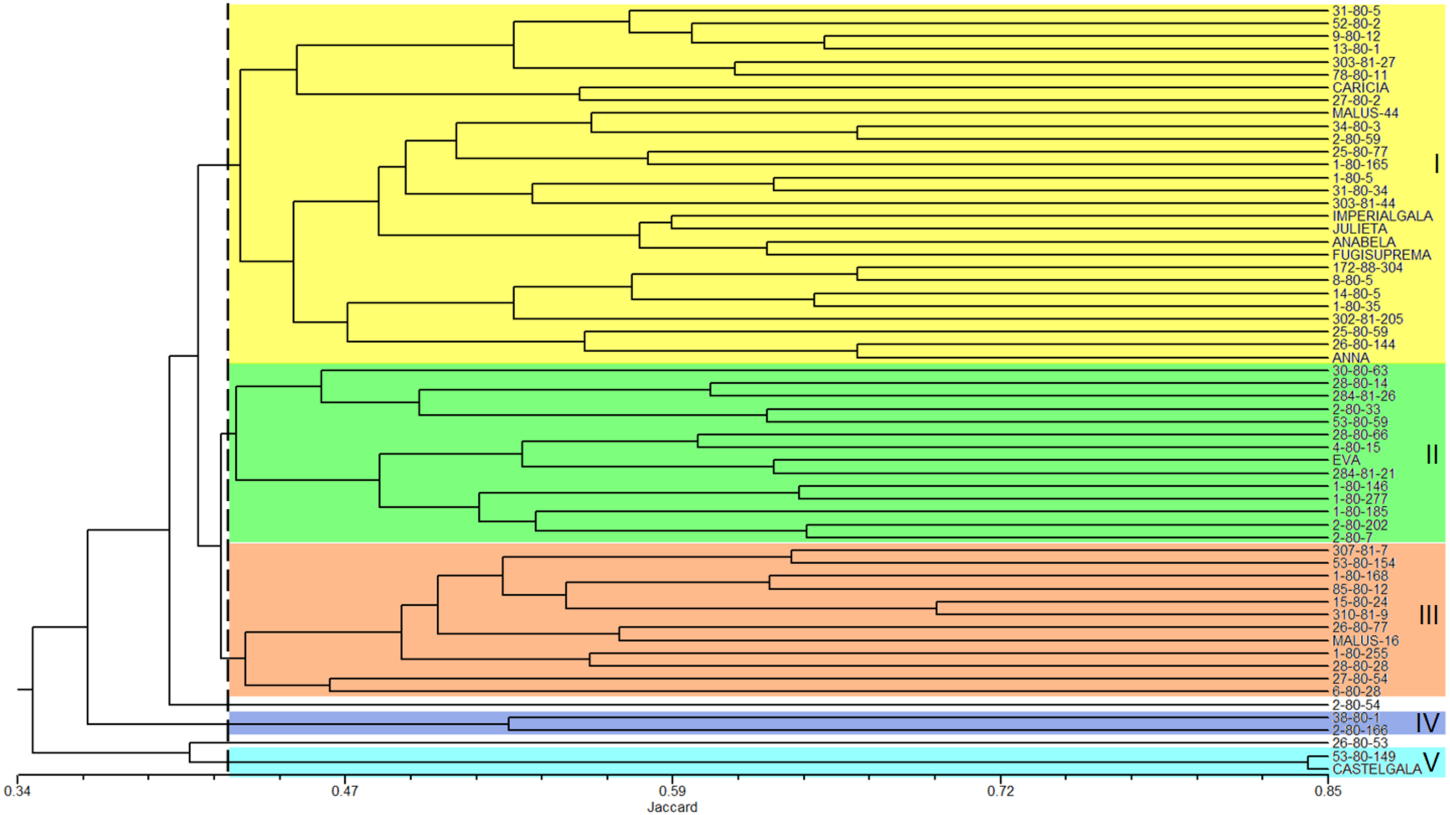
Dendrogram of the 60 apple genotypes from the IAPAR germplasm collection obtained with data from nine ISSR primers. The dotted line indicates the average similarity among all the genotypes used for cutting the dendrogram.

The analysis of the genetic similarity matrix obtained from the pairwise comparison of the 60 apple genotypes allowed us to determine that the lowest genetic similarity (20%) was between the genotypes 1-80-146 and Castel Gala and between 1-80-146 and 53-80-149, while the highest similarity (84%) was between the Castel Gala and 53-80-149 genotypes. The average similarity between all genotypes was 43%. The dendrogram resulted in the formation of five groups (I to V) and two genotypes (2-80-54 and 26-80-53) remained isolated (Fig. 3). Group I (commercial group) was formed by 28 genotypes, including most commercial cultivars; group II (EVA group) was formed by 14 genotypes, including the cultivar Eva; groups III (MALUS-16 group) and IV were formed by 12 and two genotypes, respectively and none commercial cultivar was included in these groups; the group V was formed by the genotype 53-80-149 and the commercial cultivar Castel Gala (Fig. 3).

**Figure 4:**
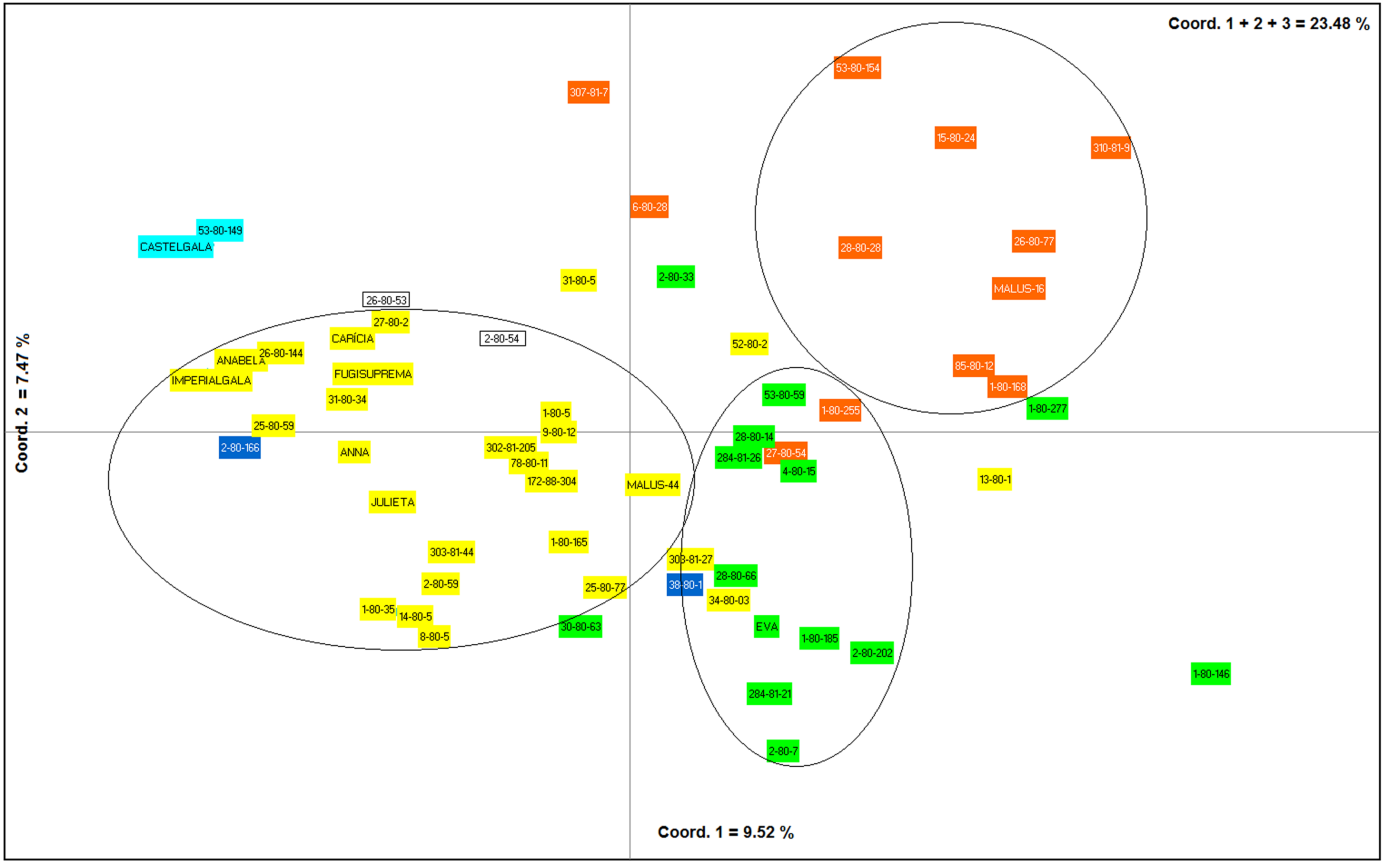
Principal Coordinates Analysis (PCoA) based on data obtained from nine ISSR primers on 60 apple genotypes from the IAPAR germplasm collection. The shaded colors of each genotype are in agreement with the colors of the groups formed in the dendrogram of Fig. 3. The circles indicate the groups formed in the PCoA.

The first, second and third axes of PCoA explain respectively 9.52, 7.47 and 6.46% of the genetic variation observed. The first three axes, cumulatively explaining 23.48% of the total genetic variation. The PCoA grouped the apple genotypes into three main clusters (Fig. 4). Most of the genotypes from group I of the dendrogram (including six of the eight commercial cultivars) were plotted in the second coordinate (Fig. 4). Already, most of the genotypes from group II (Eva group) and III of the dendrogram were plotted in the first coordinate. Genotypes from group II were most plotted in the lower half and group III in the upper half of the coordinate (Fig. 4).

**Figure 5:**
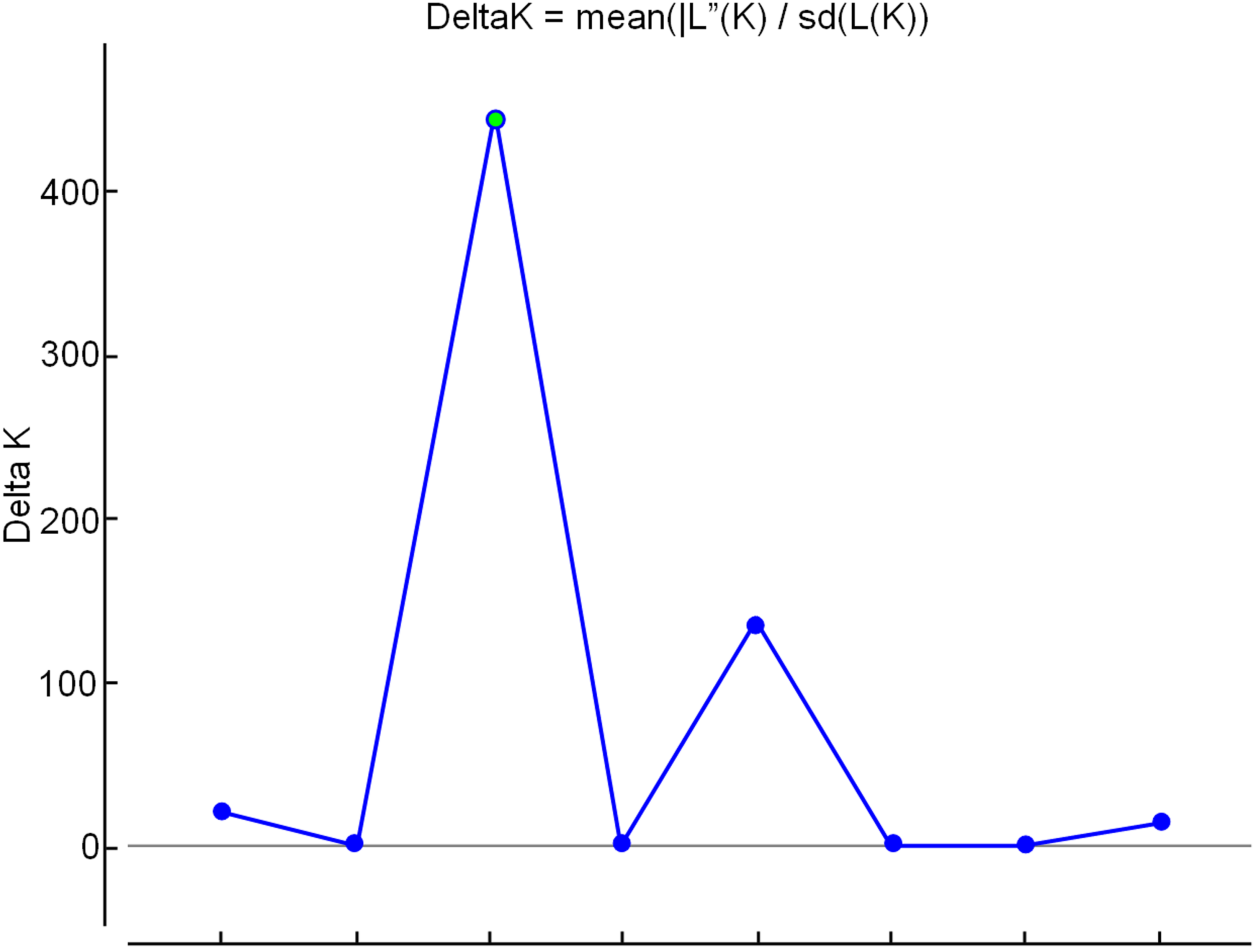
Determination of the optimal number of K (clusters-genetic groups) in apple by the Bayesian method, with data from nine ISSR markers. The point of intersection between the highest value on the *Y* axis and the *X* axis indicates the optimal number of K (genetic groups).

**Figure 6:**
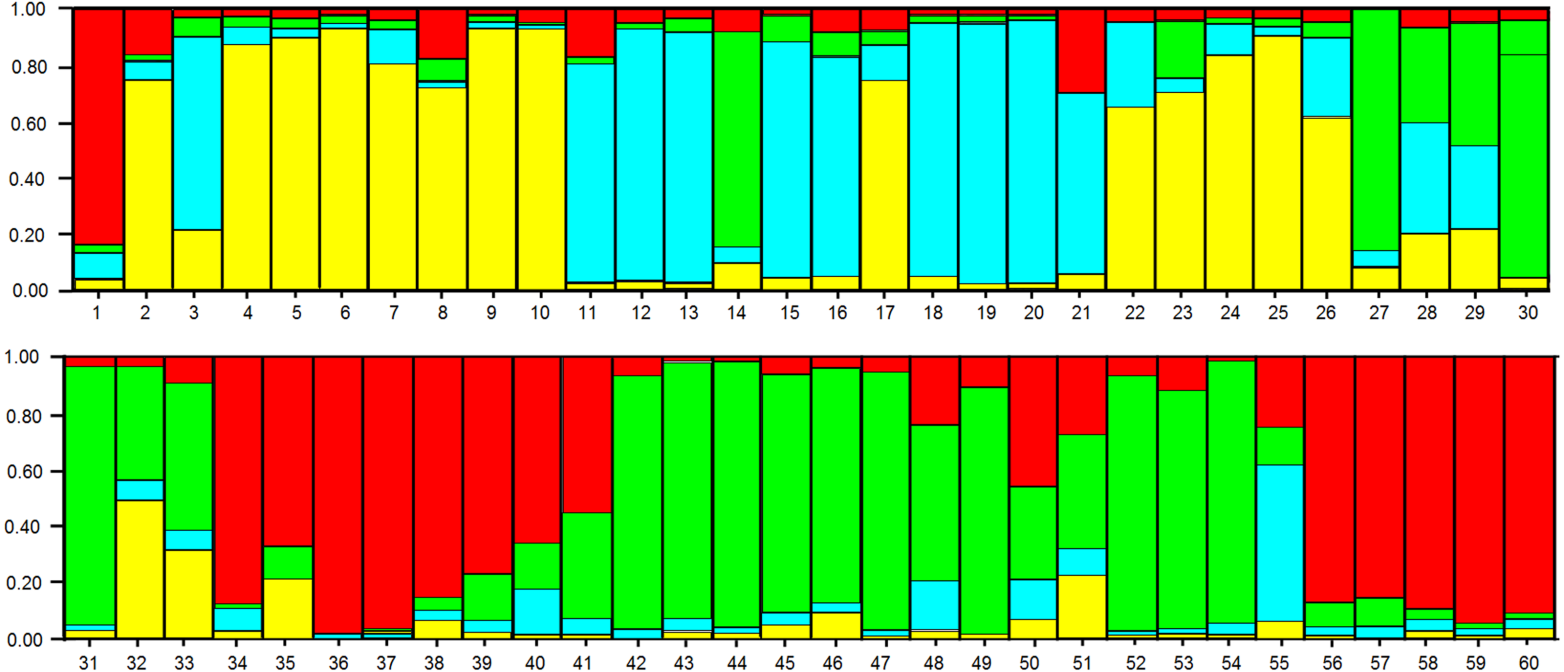
Distribution of the four genetic groups obtained by Bayesian analysis of the 60 apple genotypes from the IAPAR germplasm collection. The code of each genotype is in accordance with Table 1.

In the analysis using the Bayesian approach, the number of K (clusters, genetic groups) was defined as four (Fig. 5). The distribution of the four genetic groups among the genotypes showed the predominance of one “genetic group” in most genotypes (Fig. 6).

## Discussion

### ISSR primer selection

The selection of primers can only be considered effective when the primers selected to differentiate a species show similar results to those obtained using all available primers (Fedrigo et al., 2016). Typically, in a genetic variability study, a high number of primers is used, consequently increasing the costs and time to obtain results. Isshiki, Iwata & Khan (2008) reported that it is possible to decrease the number of primers used without losing efficiency in the results. These authors tested 100 ISSR primers for the discrimination of eight cultivars of *Solanum melongena* and concluded that 34 primers are sufficient to differentiate the genotypes with robustness.

The use of PIC and RP indexes has been effective in the selection of primers in several species, such as *Jathopha curcas* (Tatikonda et al., 2009; Grativol et al., 2011), *Ipomoea batatas* (Camargo et al., 2013), *Stenocarpella maydis* (Fedrigo et al., 2016), and *Achyrocline flaccida* (Rosa et al., 2017). Our work shows that the PIC and RP indexes successfully selected ISSR primer in apple, since the average similarity between the genotypes was the same (0.37) when using the nine selected primers and all 26 polymorphic primers. The groups formed in the dendrogram were also the same using the two sets of primers (Figs. 1A and 1B). Only the 1-80-255 genotype showed a positional change in the dendrograms however, remained in the same group (Figs. 1A and 1B). Also, maintaining bootstrapping values above 60 in the two main branches in the dendrogram (the first two nodes on the left where the groups are rooted) shows that primer selection was efficient in maintaining data reliability. The reduction in the number of primers used from 26 to nine, did not change the robustness of the results of the genetic variability study, while reducing the time and cost of the laboratory analyses by approximately 60%.

Our results show that a robust characterization of a germplasm bank is possible using approximately 40% of the resources and time that is normally spent. The strategy of primer selection to reduce costs in genetic variability analyses is the first step in democratizing the use of molecular markers in the pre-breeding step of a small plant breeding program.

### Genetic variability

When working with dominant markers such as ISSR, the percentage of polymorphisms observed can be indicative of genetic variability (Fedrigo et al., 2016). The comparison of our results with those in the literature (Table 4), shows that although we analyzed the largest number of genotypes, the average number of polymorphic fragments per primer and the general percentage of polymorphic fragments is among the smallest. However, in highly heterozygous plants such as apple, high rates of polymorphism are to be expected. Our results can be justified by the fact that the other studies have mostly been carried out by comparing different species from within the genus *Malus* or even against different or wild genetic groups. Most of the genotypes used in our work are obtained from a few crosses, and those that are from different crosses have one of the parents in common (Table 1).

**Table 4:** Results achieved in studies with ISSR markers in *Malus*.

**Authors**	**NG**	**NP**	**APFP**	**MP**
Goulão & Oliveira (2001)	41	7	32.0	89.1%
Smolik et al. (2004)	8	11	32.0	83.0%
Smolik & Krzysztoszek (2010)	8	17	7.50	69.5%
He et al. (2011)	31	20	9.95	55.2%
Pathak & Dhawan (2012)	22	15	8.90	–
This work	60	9	8.33	58.13%

**Notes.**

NG number of genotypes NP number of ISSR primers used APFP average of polymorphic fragments per primer MP mean of polymorphism

The average similarity observed among all genotypes, 43%, is below the similarities previously described for apple with ISSR markers. For example, using seven ISSR primers, Goulão & Oliveira (2001) evaluated 41 commercial apple genotypes and obtained pairwise similarities ranging from 71% to 92%. In a study on eight apple cultivars analyzed with 11 ISSR primers, the minimum similarity observed between cultivar pairs was 60% (Smolik et al., 2004). The same group, in another study, observed pairwise similarities ranging from 65 to 85% (Smolik & Krzysztoszek, 2010). In another work on *Malus*, the evaluation of 31 genotypes with 20 ISSR primers found similarity values ranging from 70% to 94% (Zhang et al., 2012). Comparing these data with those obtained in our work shows that the Brazilian apple genotypes here have a higher genetic value based on significant genotype variability that can be exploited by plant breeders. Although our data are fairly consistent, ISSR markers may be underestimating these genetic indices. In this sense, the use of molecular markers with better genome coverage, such as SNPs (single nucleotide polymorphism) may bring complementary results to those obtained here.

The combined analysis of the pedigree (Table 1) and the dendrogram (Fig. 3) does not show a clear relationship between dendrogram groups and pedigree, since the genotypes derived from Anna are in different groups. The dendrogram shows that most commercial cultivars (six cultivars of the eight studied) were in the same group (Fig. 3). This grouping is probably reflecting the similarity that these cultivars share related to the market and agronomic characteristics selected by the breeders during their development. Eva is the most important apple cultivar for regions with warmer climates (Castro et al., 2015; Pommer & Barbosa, 2009). Interestingly, this cultivar was genetically distant from the other commercial cultivars, perhaps reflecting some unique characteristics that makes it with great performance in warmer climates. In this sense, the other genotypes of the Eva Group in the dendrogram are the most promising to be considered in the development of cultivars for climates similar to those that Eva has a good performance.

The PCoA (Fig. 4) corroborate the results obtained in the dendrogram (Fig. 3). The same groups observed in the dendrogram (commercial group, Eva group and MALUS-16 group) were observed in PCoA. These results evidenced the robustness of ISSR markers in understanding the distribution of genetic variability in apple. The scattering of genotypes across all quadrants of the PCoA and the poor explanation of the genetic variation by the three first coordinates (Fig. 4) shows the high variability of the IAPAR apple germplasm collection and that this variability can not be explained by few genotypes. This high genetic variability was corroborated by the Bayesian analysis that indicated the presence of five genetic clusters in this apple collection (Fig. 6). Therefore, we conclude that the variability is not restricted to a specific group of genotypes but is rather distributed throughout all genotypes (Fig. 6).

*Malus*, as many other species in the family Rosaceae, shows gametophytic self-incompatibility, which forces outcrossing ( Pereira-Lorenzo et al., 2018). As a consequence, the species has a high genetic variability and diversity. In this sense, when performing crosses there is a very high segregation in the progeny and this makes the progeny very differentiated by carrier different allelic combinations. A detailed analysis of Table 1 shows that among the studied genotypes there are several ones obtained from crosses involving the cultivar Anna and others cultivars with different market characteristics (shape, color, consistency, flavor, sweetness, acidity). Until now it was not known which of these genotypes were genetically closest to Eva, one of the most cultivated apple in warm regions. Our data show that genotypes 1-80-184, 28-80-66, 2-80-202, 284-81-20, and 2-80-7 were the genetically closest to Eva in the dendrogram (Fig. 3) as in PCoA (Fig. 4). Interestingly, only two of these genotypes (2-80-202 and 2-80-7) originate from the same cross that originated Eva (Anna × Gala) (Table 1). The other three genotypes were obtained from crosses involving Anna and other cultivars with different phenotypic characteristics of the cultivar Gala. These data show the potential in the IAPAR apple germplasm collection for the development of cultivars with different market characteristics (shape, color, consistency, flavor, sweetness, acidity) of Eva and with low chilling requirements.

### Conclusion

Genetic variability is the basis of a plant breeding program (Marić et al., 2010; Morales et al., 2011; Camargo et al., 2013; Morales et al., 2013). Therefore, the IAPAR apple genotype collection represents a resource with a high potential for obtaining superior cultivars, since it is characterized by high genetic variability. It is also notable that, in this collection, there are important cultivar sources for improvements based on low chilling requirements, resistance to scab and variable market characteristics (flavor, sweetness, acidity, consistency, color) characteristics. Thus, our data on variability and grouping of the genotypes, together with data from the field, could help breeders to select genotypes for crosses to develop new cultivars adapted to the current tropical climatic conditions, as well as those conditions that may arise due to global warming.

##  Supplemental Information

10.7717/peerj.6265/supp-1 Supplemental Information 1 Apple binary data Raw data with the binary data obtained with the reading of the gels obtained with the nine ISSR primers in the 60 apple genotypes.

10.7717/peerj.6265/supp-2 Supplemental Information 2 ISSR gels Raw data with gels obtained with the nine ISSR primers in the 60 apple genotypes.

## Additional Information and Declarations

### Data Availability

The following information was supplied regarding data availability: Raw data are available in the Supplemental Files.
